# Occurrence and analysis of mycotoxins in domestic Chinese herbal medicines

**DOI:** 10.1080/21501203.2020.1727578

**Published:** 2020-02-20

**Authors:** Lu Qin, Jia-Yi Jiang, Lei Zhang, Xiao-Wen Dou, Zhen Ouyang, Li Wan, Mei-Hua Yang

**Affiliations:** a Key Laboratory of Bioactive Substances and Resources Utilization of Chinese Herbal Medicine, Ministry of Education, Institute of Medicinal Plant Development, Chinese Academy of Medical Sciences & Peking Union Medical College, Beijing, China; b Pharmacy College, Chengdu University of Traditional Chinese Medicine, Chengdu, China; c School of Pharmacy, Jiangsu University, Zhenjiang, China

**Keywords:** Chinese herbal medicine, mycotoxin, maximum residue limits, detection method, contamination

## Abstract

For time immemorial, Chinese herbal medicines (CHMs) have been widely used in China for disease treatment and promotion of general well-being. However, in recent years, many studies have shown that mycotoxins produced by fungi could contaminate CHMs due to unfavourable pre- or post-harvest conditions, raising major concern for consumer safety. At present, there is a significant focus on developing novel mycotoxin detection methods for analysing CHMs, and numerous studies have aimed to determine which kinds of raw herbal materials are most susceptible to mycotoxin contamination. In this review, we focus on recent advances in understanding and detection of mycotoxins in domestic raw herbal materials and related products from 2000 to 2018. Aspects of mycotoxin contamination of CHMs covered in this review include common mycotoxin contaminants in CHMs, maximum mycotoxin residue limits, analytical methods for mycotoxin detection and their applications and limitations, as well as a brief discussion of the trends in ongoing research.

## 1. Introduction

Mycotoxins are secondary metabolites produced by fungi during growth that can cause pathological responses in humans and animals. Medicinal herbs are highly susceptible to toxigenic fungal infections and mycotoxin contamination that can occur at either the pre- or post-harvest stage as a result of poor growing conditions, inadequate drying, or storage in warm, humid conditions (Zhang et al. 2016). The potential for medicinal herbs and related agricultural products to have toxic effects as a result of mycotoxin contamination is attracting increasing attention worldwide (Tripathy et al. 2015; Mahfuz et al. 2018; Zhang et al. 2018c). At present, the most common mycotoxins found in Chinese herbal medicines (CHMs) are aflatoxins, ochratoxins, zearalenone, fumonisins, trichothecenes, and patulin (Zhang et al. 2015). Numerous studies have shown that these mycotoxins are highly toxic resulting in hepatotoxicity, nephrotoxicity, reproductive disorders, and immuno-suppression. These mycotoxins are also carcinogenic, teratogenic, and mutagenic making exposure to compounds of this nature a serious human health threat.

## 2. Aflatoxins (AFs)

In 1960, 100,000 turkeys died abruptly over the span of a few months in the UK. People later found that they had all consumed the same peanut meal that was contaminated by fungi. As a result of this occurrence, AFs were discovered and characterised (Wannop 1961). AFs are secondary metabolites that share a common difurocoumarin skeleton; they are produced by *Aspergillus flavus* and *A. parasiticus* (Shen et al. 2016). This class of compounds includes aflatoxin B_1_, B_2_, G_1_, G_2_, M_1_, and M_2_; of these, AFB_1_ is the most toxic and carcinogenic one. According to previous studies, the toxicity of AFB_1_ is 10 times greater than that of cyanide and 68 times greater than that of arsenic. In 1993, AFB_1_ was classified as a Class 1A carcinogen by the World Health Organisation Cancer Research Institute (Ono et al. 2001). Studies have shown that AFB_1_ can suppress the immune system and affect foetal development and differentiation of cells, giving this compound the ability to exert teratogenic effects. Exposure to AFB_1_ is also known to have damaging effects on human and animal liver tissues. In severe cases, exposure to AFB_1_ resulted in liver cancer and even death (Ma and Zan 2009).

### 2.1. The limit standards of AFs

By the end of 2003, approximately 100 countries had imposed specific limits on the levels of mycotoxins allowed in food and feed (Food and Agriculture Organization of the United Nations 2004). As depicted in Table 1, in the case of medicinal herbs and related products, the legal limit for AFB_1_ ranges from 2 to 10 μg∙kg^−1^, while the limit for other AFs ranges from 4 to 20 μg∙kg^−1^. Among these regulations, the European Pharmacopeia (European Pharmacopoeia Commission 2016) and the British Pharmacopeia (British Pharmacopoeia Commission 2012) have set the strictest limits (2 μg∙kg^−1^ for AFB_1_ and 4 μg∙kg^−1^ for total AFs), and the most commonly set limits for AFB_1_ and AFs are similar to those of the Chinese Pharmacopeia (Chinese Pharmacopoeia Commission 2015) and the European Union (European Union 2006), which were 5 μg∙kg^−1^ and 10 μg∙kg^−1^, respectively.

**Table 1.** Limits of AFs for medicinal plants in Standards and Regulations.

Standards and regulations	Product (Group)	AFB_1 (_µg∙kg^−1^)	Total AFs (µg∙kg^−1^)	Reference
EU	Nutmeg			(European Union 2006)
	Ginger	5	10	
	Turmeric			
	White and black pepper			
EP	Herbal drugs	2	4	(European Pharmacopoeia Commission 2016)
USP	Some types of raw medicinal herb materials, as well as their powder and/or dry extract	5	20	(United States Pharmacopeial Convention 2017)
BP	Herbal drugs	2	4	(British Pharmacopoeia Commission 2012)
Canada	Products containing ginseng or any substance derived from this source, Evening Primrose Oil, sugar cane, sugar beets, cottonseed	5	20	(Government of Canada, Natural and Non-prescription Health Products Directorate 2015)
JP	Crude drug and preparations containing crude drugs as main ingredient (crude drug preparations)		10	(Japanese Pharmacopoeia Commentary Editorial Committee 2016)
KP	*Armeniacae Semen, Arecae Semen, Cassiae Semen, Crotonis Semen, Curcumae Radix, Dolichoris Semen, Glycyrrhizae Radix et Rhizoma, Nelumbinis Semen, Myristicae Semen, Persicae Semen, Pinelliae Tuber, Polygalae Radix, Carthami Flos, Thujae Semen, Trichosanthis Semen, Zizyphi Semen*	10	15	(Korean Food & Drug Administration 2012)
ChP	*Bombyx Batryticatus, Citri Reticulatae Pericapium, Sterculiae Lychnophorae Semen, Persicae Semen, Ziziphi Spinosae Semen, Platycladi Semen, Nelumbinis Semen, Quisqualis Fructus, Arecae Semen, Hordei Fructus Germinatus, Myristicae Semen, Cassiae Semen, Polygalae Radix, Coicis Semen, Jujubae Fructus, Phenetima, Scolopendra, Hirudo, Scorpio*	5	10	(Chinese Pharmacopoeia Commission 2015)
HKCMMS	Herbal drugs	5	10	(Department of Health, Hong Kong Special Administrative Region of the People’s Republic of China 2005)
Indonesia	Coconut, spices and traditional drug medicines/herbs		20	(Food and Agriculture Organization of the United Nations 2004)
Vietnam	Nutmeg Ginger and turmeric Black and white pepper Liquorice root used for herbal tea Liquorice extract for beverage or to mix	5	10	(United States Department of Agriculture Foreign Agricultural Service 2013)
Germany	Any materials used in manufacture of medicinal products (including medicinal herbal products)	2	4	(World Health Organization. 2007)
Argentina	Herbs, herbal materials and herbal preparations used for herbal tea infusions	5	20	(World Health Organization (WHO) 2007)

### 2.2. Detection methods for AFs

Thin-layer chromatography (TLC) was the first method used for detecting AFs. In correlation with the increasing demand for more precise data, the overall percentage of use of TLC for detecting AFs was reduced. However, since TLC is a straightforward approach with low-associated cost and minimal specialised equipment, it is still generally utilised in some laboratories (Li et al. 2005).

In recent years, high-performance liquid chromatography (HPLC) has become the most common method for quantifying AFs. Currently, HPLC with an FLD detector (HPLC-FLD) is the most commonly used method for detecting the presence of AFs and quantifying their levels. However, aqueous solvents are often used as the eluent in reverse-phase chromatography, and aqueous buffers will partially quench the fluorescence of AFB_1_ and AFG_1_. Therefore, a derivatisation step is usually required to stabilise and enhance detection, such as a pre-column derivatisation with trifluoroacetic acid (TFA) (Zhao et al. 2011; Li et al.^2015^) or a post-column derivatisation such as a chemical derivatisation with iodine or bromine (Ran et al. 2017), a photochemical derivatisation, or an electrochemical derivatisation (Zhang et al. 2005a; Zhang and Chen 2005). Compared to pre-column derivatisations, application of post-column derivatisations was reported more frequently (Zhang et al. 2018c).

HPLC-MS/MS has been increasingly used for the detection and quantification of AFs in herbal medicines (Wang et al. 2011; Liu et al. 2012; Saha et al. 2018). At the same time, HPLC-MS/MS is often used for confirmation of AF identity in order to avoid interfering signals from analogs of AFs that might be present in herbal medicines.

In addition to conventional analysis methods, immunological methods have been used for rapid detection of AFs in CHMs such as an enzyme-linked immunosorbent assay (ELISA) and a gold immunochromatographic assay (GICA). Chu *et al*. (Chu et al. 2015) used these approaches to successfully detect AFB_1_ in lotus seeds. Since the complexity matrices presented by CHMs can affect the antigen-antibody specific binding reaction, a matrix-matching curve was used to reduce the bias introduced by the matrix. For example, using AFB_1_-BSA and a sheep anti-mouse IgG antibody for detection and a control, respectively, Yang generated a test strip suitable for rapid detection of AFB_1_ in lotus seeds with a sensitivity of 2.5 ng∙mL^−1^ (Yang 2015).

Fluorescent immunoassays (FIAs) have recently been developed for qualitative and quantitative analysis of AFs in herbal matrices. Zhang established a fluorescence polarisation immunoassay detection method by combing AFB_1_ with amino fluorescein. The molecular weight and rotation speed of the AFB_1_ fluorescent probe will change before and after binding with an antibody. Thus, detection and quantification of AFB_1_ in CHMs can be accomplished by measuring the change in the fluorescence polarisation value (Zhang 2017). Based on the development of a FITC-AFB_1_ fluorescently labelled antibody, Yu et al. established a direct competitive fluorescent immunoassay to detect AFB_1_ in five CHMs (Yu et al.^2015^). Zhang utilised PEG-modified CdSe/CdS quantum dots (QDs) with glycine-based signal enhancement for the detection of AFB_1_ in medicinal herbs (Zhang et al. 2018b). This work demonstrated that a QD labelling technique could potentially serve as a novel means of performing fast trace-detection in complex herbal matrices. Due to the AFB_1_ self-sensitisation to fluorescence when under UV light, a label-free FIA method was developed for the specific detection of AFB_1_ in CHMs. Compared with traditional immunoassay approaches, this method could reduce the cost of analysis and shorten the analysis time without a complex probe labelling process (Shu 2018).

### 2.3. AF contaminants in CHMs

**Figure 1.**
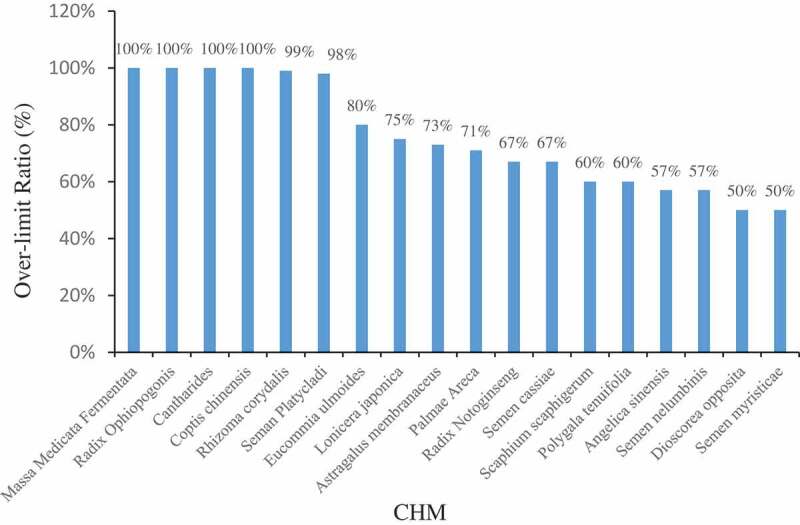
CHM with AFB_1_ exceeding the standard rate of 50%Note: AFB_1_ limit standard was 5 μg∙kg^−1^.

From 2000 to 2018, 2979 batches of CHMs from 66 varieties known to be easily contaminated by AFB_1_ were tested, as summarised in Table 2, of which 697 batches tested positive for AFB_1_. Levels of AFB_1_ ranged from 0.02 to 1268.8 μg∙kg^−1^. It is important to note that the percentage of the botanicals *Zingiber officinale* (Kuang 2000; Bao et al. 2008; Cao 2013) and *S. Platycladi* (Yang et al. 2004, 2005; Yang et al. 2011b; Hao et al. 2012; Hu et al. 2012; Liu et al. 2012a; Hao et al. 2013; Li 2015a; Zhu et al. 2015; Chien et al. 2018) that tested positive for AFB_1_ was 68% and 78%, respectively. In the case of the animal material *Cantharides*, the per cent positive rate for contamination with AFB_1_ was as high as 95%, and the lowest contaminating amount detected was 25.95 μg∙kg^−1^; this is more than 5 times the limit set by the EU and China (5 μg∙kg^−1^), indicating that these types of samples are highly susceptible to AFB_1_ contamination (Sun and Liu 2016). In addition, of the CHM samples that tested positive, there were 486 batches with AFB_1_ levels exceeding the limits set by the EU and China, accounting for 70% of the total positive samples. CHMs with a per cent positive for AFB_1_ of above 50% included *Massa Medicata Fermentata* and *R. Ophiopogonis* (Figure 1).

**Table 2.** Detection of AFs in CHM.

	AFB_1_					Afs (AFB_1_+ AFB_2_+ AFG_1_+ AFG_2_+ AFM_1_)					
CHM	Total samples	Positive samples No(%)	Range (µg∙kg^−1^)	>EP/BP Legal limit No (%)	>Ch.P/EU Legal limit No (%)	Total samples	Positive samples No (%)	Range (µg∙kg^−1^)	>EP/BP Legal limit No (%)	>Ch.P/EU Legal limit No (%)	Reference
*Panax Ginseng*	49	11(22%)	0.02–5.81	5(10%)	2(4%)	41	10(24%)	0.14–11.92	4(10%)	2(5%)	(Kuang 2000; Liang and Huang 2000; Bao et al. 2008; Zheng et al. 2010b; Zhao et al. 2011; Zheng et al. 2014c; Lin et al. 2016; Chien et al. 2018)
*Dioscorea opposita*	95	10(11%)	0.03–74.84	5(5%)	5(5%)	67	3(4%)	0.7–1.1	0	0	(Kuang 2000; Liang and Huang 2000; Tang 2000; Hao et al. 2012; Wang et al. 2014b; Zheng et al. 2014c; Li 2016; Lin et al. 2016; Chien et al. 2018; Zhang et al. 2018a)
*Rheum officinale*	8	5(45%)	0.38–187.25	2(25%)	2(25%)	3	1(33%)	3.3	0	0	(Kuang 2000; Liang and Huang 2000; Tang 2000; Zhang et al. 2005; Zhao et al. 2011; Hao et al. 2012; Lin et al. 2016)
*Atractylodes macrocephala*	16	4(25%)	0.17–15	1(6%)	1(6%)	12	2(17%)	0.54	0	0	(Kuang 2000; Liang and Huang 2000; Zhang et al. 2005; Hao et al. 2012; Zheng et al. 2014c; Lin et al. 2016)
*Pinellia ternata*	4	3(75%)	0.04–1.89	0	0	4	3(75%)	0.04–1.89	0	0	(Kuang 2000; Liang and Huang 2000; Hu et al. 2012)
*Salvia miltiorrhiza*	8	3(38%)	1.21–3.46	1(13%)	0	6	0	0	0	0	(Kuang 2000; Zhang et al. 2005; Zheng et al. 2005; Yang et al. 2013)
*Lonicera japonica*	11	4(36%)	1.96–50.00	3(27%)	3(27%)	6	3(50%)	0.95–203	2(33%)	2(33%)	(Kuang 2000; Li and Zhuang 2000; Tang 2000; Zhang et al. 2005; Hao et al. 2012; Tan et al. 2012)
*Fructus Crataegi*	65	3(5%)	0.12–28	1(2%)	1(2%)	62	0		0	0	(Kuang 2000; Zheng et al. 2005; Li et al. 2011a, Li et al. 2012; Zhang et al. 2008; Zhu et al. 2015; Chien et al. 2018)
*Zingiber officinale*	31	21(68%)	0.03–8.88	9(29%)	5(16%)	30	20(67%)	0.05–22.06	6(20%)	4(13%)	(Kuang 2000; Bao et al. 2008; Cao 2013)
*Massa Medicata Fermentata*	52	6(12%)	0.38–29.38	6(12%)	6(12%)	47	8(17%)		1(2%)	1(2%)	(Li and Zhuang 2000; Li and Chen 2000; Tang 2000; Yang et al. 2004; Zhang et al. 2005; Zhu et al. 2015; Chien et al. 2018)
*Radix Paeoniae alba*	78	7(9%)	0.78–3.44	3(4%)	0	77	8(10%)	0.47–7.82	1(1%)	0	(Li and Chen 2000; Yang et al. 2013; Li 2016; Xing et al. 2016; Chien et al. 2018; Zhang et al. 2018a)
*Astragalus membranaceus*	228	11(5%)	0.07–200	9(4%)	8(4%)	225	10(4%)	0.38–64.3	9(4%)	3(1%)	(Li and Chen 2000; Liang and Huang 2000; Han et al. 2012; Zheng et al. 2010b; Tan et al. 2012; Yang et al. 2013; Lin et al. 2016; Chien et al. 2018)
*Pericarpium citri reticulatae*	181	19(10%)	0.12–118.5	9(5%)	5(3%)	175	35(20%)	0.03–77.55	6(3%)	5(3%)	(Li and Zhuang 2000; Li and Chen 2000; Liang and Huang 2000; Tang 2000; Zhang et al. 2005; Zheng et al. 2010b; Li et al. 2011a; Yang et al. 2011a; Wang et al. 2012; Yang et al. 2013; Li et al. 2014b; Wang et al. 2014b; Wang et al. 2014c; Zhu et al. 2015; Lin et al. 2016; Chien et al. 2018)
*Radix bupleuri*	6	2(33%)	10–26.85	2(33%)	2(33%)	4	0		0	0	(Li and Zhuang 2000; Li and Chen 2000; Hao et al. 2012; Yang et al. 2013)
*Cortex Moutan*	3	1(33%)	2.5	1(33%)	0	2	0		0	0	(Li and Chen 2000; Hao et al. 2012)
*Radix isatidis*	5	3(60%)	0.2–100	2(40%)	2(40%)	2	0		0	0	(Li and Chen 2000; Liang and Huang 2000; Tang 2000; Yang et al. 2013)
*Scutellaria baicalensis*	5	2(40%)	16.9–22.82	2(40%)	2(40%)	3	0		0	0	(Li and Zhuang 2000; Li and Chen 2000; Zhang et al. 2005; Hao et al. 2012)
*Radix Ophiopogonis*	20	3(15%)	34.12–89.96	3(15%)	3(15%)	15	1(7%)	1.89	0	0	(Li and Chen 2000; Tang 2000; Hao et al. 2012; Hu et al. 2012; Liu et al. 2012a; Yang et al. 2013)
*Scaphium scaphigerum*	42	5(12%)	1.0–8.378	4(10%)	3(7%)	40	3(8%)	1–10.546	2(5%)	1(3%)	(Li and Chen 2000; Yang et al. 2004; Yang et al. 2005; Zhang et al. 2005; Zheng et al. 2010b; Su et al. 2014; Wang et al. 2014c; Zhu et al. 2015; Lin et al. 2016)
*Fructus Aurantii Immaturus*	4	4(100%)	0.13–107.08	3(2%)	3(2%)						(Li and Chen 2000; Liang and Huang 2000; Tang 2000)
*Poria cocos*	46	6(13%)	0.05–6.12	2(4%)	2(4%)	34	3(9%)	0.758–6.12	1(3%)	0	(Li and Chen 2000; Liang and Huang 2000; Zhang et al. 2005; Zhao et al. 2011; Xie 2016; Chien et al. 2018; Hu et al. 2018)
*Polygonum cuspidatum*	3	1(5%)	0.1	0	0	1	1(100%)	6.8	1(100%)	0	(Liang and Huang 2000; Guo et al. 2012; Han et al. 2012; Lin et al. 2016)
*Rhizoma rehmanniae*	5	4(33%)	0.17–85.46	3(60%)	2(40%)	1	0		0	0	(Li and Zhuang 2000; Liang and Huang 2000; Tang 2000; Han et al. 2012; Lin et al. 2016)
*Semen Ziziphi Spinosae*	42	17(40%)	0.4–23.4	7(17%)	4(10%)	38	15(39%)	0.4–25.6	5(13%)	4(11%)	(Liang and Huang 2000; Zhang et al. 2008; Han et al. 2010; Zheng et al. 2010b; Zheng et al. 2010b; Li et al. 2011; Yang et al. 2011a; Hao et al. 2012; Hu et al. 2012; Yang et al. 2013; Wang et al. 2014b; Zhu et al. 2015; Lin et al. 2016; Chien et al. 2018)
*Radix scrophulariae*	5	1(20%)	0.03	0	0	3	1(33%)	4.4	1(33%)	0	(Liang and Huang 2000; Guo et al. 2012; Hao et al. 2012; Lin et al. 2016)
*Platycodon grandiflorum*	5	1(20%)	0.16	0	0	3	0		0	0	(Liang and Huang 2000; Zhang et al. 2005; Hao et al. 2012; Lin et al. 2016)
*Schisandra chinensis*	25	2(8%)	0.11–0.54	0	0	22	0		0	0	(Liang and Huang 2000; Zhang et al. 2008; Yang et al. 2013; Lin et al. 2016; Chien et al. 2018)
*Codonopsis pilosula*	8	1(13%)	28.48	1(13%)	1(13%)	6	1(17%)	471	1(17%)	1(17%)	(Li and Zhuang 2000; Li and Chen 2000; Yang et al. 2004; Hao et al. 2012; Tan et al. 2012; Lin et al. 2016)
*Polygala tenuifolia*	20	10(50%)	0.01–118.1	9(45%)	6(30%)	20	10(50%)	0.01–118.1	6(30%)	6(30%)	(Liang and Huang 2000; Hao et al. 2012, 2013; Chien et al. 2018)
*Radix glycyrrhizae*	80	25(31%)	0.16–112.79	7(9%)	7(9%)	75	23(31%)	0.027–26.11	4(5%)	3(4%)	(Liang and Huang 2000; Tang 2000; Zhang et al. 2005; Wei et al. 2011; Zhao et al. 2011; Guo et al. 2012; Tan et al. 2012; Li et al. 2014b; Lin et al. 2016)
*Semen Armeniacae Amarae*	51	11(22%)	0.06–9.81	6(12%)	1(2%)	49	10(20%)	0.06–11.95	2(4%)	1(2%)	(Liang and Huang 2000; Yang et al. 2004; Yang et al. 2005; Zhang et al. 2005; Zheng et al. 2005; Zhang et al. 2008; Han et al. 2010; Li et al. 2011a; Han et al. 2012; Li et al. 2012; Liu et al. 2012a; Tan et al. 2012; Zheng et al. 2013; Wang et al. 2014b; Zhu et al. 2015; Zhao et al. 2016)
*Polygonum multiflorum*	59	3(5%)	0.06–6.8	2(3%)	1(2%)	57	22(39%)	2.1–25	4(7%)	2(4%)	(Liang and Huang 2000; Han et al. 2012; Guo et al. 2015; Li et al. 2016; Lin et al. 2016)
*Eucommia ulmoides*	9	5(56%)	0.85–61.42	4(44%)	4(44%)	5	1(20%)	29.85	1(20%)	1(20%)	(Tang 2000; Hao et al. 2012; Tan et al. 2012)
*Semen Sojae Praeparatum*	6	2(33%)	21.52–124.2	2(33%)	2(33%)	4	0		0	0	(Li and Zhuang 2000; Tang 2000; Zhang et al. 2005; Zheng et al. 2005; Hao et al. 2012; Wang et al. 2014b)
*Coptis chinensis*	5	3(60%)	15.71–68.5	3(60%)	3(60%)	2	0		0	0	(Tang 2000; Hao et al. 2012)
*Angelica sinensis*	41	7(17%)	0.4–121.62	6(15%)	4(10%)	37	2(5%)	4.45–4.91	2(5%)	0	(Li and Zhuang 2000; Tang 2000; Zheng et al. 2010b; Hao et al. 2012; Tan et al. 2012; Yang et al. 2013; Liu et al. 2015)
*Rhizoma Gastrodiae*	15	4(27%)	0.758	0	0	1					(Tang 2000; Zhang et al. 2005; Hu et al. 2012; Xie 2016)
*Semen Coicis*	208	25(12%)	0.09–45.3	7(3%)	5(2%)	207	48(23%)	0.10–12.4	5(2%)	5(2%)	(Yang et al. 2004; Yang et al. 2005; Zhang et al. 2008; Zheng et al. 2010b; Li et al. 2011a; Zhao et al. 2011; Hao et al. 2012; Li et al. 2012; Kong et al. 2013; Wang et al. 2014b; Zheng et al. 2014b; Zhu et al. 2015; Zhao et al. 2016; Chien et al. 2018)
*Fructus hordei germinatus*	73	13(18%)	0.42–20.5	5(7%)	3(4%)	51	8(16%)	0.47–9.46	1(2%)		(Li and Zhuang 2000; Yang et al. 2004; Yang et al. 2005; Zhang et al. 2005; Zhang et al. 2008; Wang et al. 2014b; Zheng et al. 2014c; Li 2015a; Zhu et al. 2015; Wang 2016; Wang et al. 2016; Zhang et al. 2018a)
*Seman Platycladi*	279	217(78%)	0.25–592.0	214(77%)	212(76%)	279	257(92%)	1.35–135.7	191(68%)	190(68%)	(Yang et al. 2004; Yang et al. 2005; Yang et al. 2011b; Hao et al. 2012; Hu et al. 2012; Liu et al. 2012a; Hao et al. 2013; Li 2015a; Zhu et al. 2015; Chien et al. 2018)
*Radix Gentianae Macrophyllae*	3	1(33%)	34.43	1(33%)	1(33%)	3	2(67%)	0.6757–34.43	1(33%)	1(33%)	(Li and Zhuang 2000; Yang et al. 2004; Zhang et al. 2005)
*Semen Persicae*	72	14(19%)	0.34–35.94	6(8%)	3(4%)	72	14(19%)	0.42–35.94	5(7%)	1(1%)	(Li and Zhuang 2000; Yang et al. 2004; Yang et al. 2005; Han et al. 2010; Zheng et al. 2010b; Li et al. 2011a; Yang et al. 2011a; Han et al. 2012; Hao et al. 2012; Li et al. 2012; Li et al. 2014b; Wang et al. 2014b; Zheng et al. 2014a; Zhu et al. 2015; Chien et al. 2018)
*Semen cassiae*	14	6(43%)	0.55–12.9	5(36%)	4(29%)	14	7(50%)	0.70–7.49	2(14%)	2(14%)	(Zhang et al. 2005; Zheng et al. 2005; Guo et al. 2012; Hao et al. 2012; Liu et al. 2012a)
*Radix sophorae flavescentis*	4	1(25%)	27.6	1(25%)	1(25%)	4	1(25%)	27.6	1(25%)	1(25%)	(Zhang et al. 2005; Guo et al. 2012; Hao et al. 2012, 2013)
*Radix angelicae*	14	3(21%)	0.743	0	0	3					(Zhang et al. 2005; Hao et al. 2012; Hu et al. 2012; Xie 2016)
*Fructus Ziziphi Jujubae*	151	8(5%)	1.2–4.67	2(1%)	0	151	10(7%)	0.7–7.7	3(2%)		(Zheng et al. 2005; Liau et al. 2010; Zheng et al. 2010b; Li et al. 2011a; Guo et al. 2012; Liu et al. 2012a; Zhu et al. 2015; Zhao et al. 2016; Chien et al. 2018)
*Bombyx Batryticatus*	48	14(29%)	0.23–39	6(13%)	5 (10%)	47	15(32%)	0.38–42	6(13%)	6(13%)	(Zheng et al. 2005; Zheng et al. 2010b; Yang et al. 2011a; Yang et al. 2011c; Li et al. 2014b; Wang et al. 2014c; Lin et al. 2016; Liu et al. 2017; Luo et al. 2018)
*Cantharides*	21	20(95%)	25.95–295.73	20(95%)	20(95%)	21	20(95%)	43.47–301.87	20(95%)	20(95%)	(Sun and Liu 2016)
*Semen nelumbinis*	189	60(32%)	0.21–1268.8	35(19%)	34(18%)	169	46(27%)	0.21–1268.8	25(15%)	23(14%)	(Li et al. 2011a, 2012; Liu et al. 2012; Liu et al. 2013; Zheng et al. 2014c; Chu et al. 2015; Wang 2016; Zhao et al. 2016; Chien et al. 2018; Zhang et al. 2018a)
*Hirudo*	14	2(14%)	1.26–3.14	1(7%)	0	14	2(14%)	1.26–3.35			(Yang et al. 2011c; Liu et al. 2017; Luo et al. 2018)
*Eupolyphaga seu Steleophaga*	36	17(19%)	0.33–28.81	11(31%)	8(22%)	36	21(58%)	1.1–257.55	14(39%)	11(31%)	(Yang et al. 2011c; Liu et al. 2017; Sun et al. 2017; Luo et al. 2018)
*Ligusticum wallichii*	2	1(50%)	2.547	1(50%)	0	1					(Hao et al. 2012; Hu et al. 2012)
*Semen plantaginis*	3	1(33%)	0.39	0	0	2					(Zhang et al. 2008; Hao et al. 2012; Liu et al. 2012a; Chien et al. 2018; Zhang et al. 2018a)
*Palmae Areca*	34	17(50%)	0.06–97.1	12(35%)	10(29%)	34	19(56%)	0.21–97.1	8(24%)	7(21%)	(Li et al. 2011a; Hao et al. 2012; Li et al. 2012; Hao et al. 2013; Li 2015a)
*Rhizoma corydalis*	276	78(28%)	0.21–693.4	77(28%)	77(28%)	276	117(42%)	1.01–693.4	65(24%)	64(23%)	(Yang et al. 2005; Hao et al. 2012; Liu et al. 2012a; Hao et al. 2013; Chien et al. 2018)
*Rhizoma seu Radix Notopterygii*	2					2	1(50%)	41.5	1(50%)	1(50%)	(Guo et al. 2012; Hao et al. 2012)
*Semen myristicae*	15	6(40%)	0.13–239.62	4(27%)	3(20%)	15	6(40%)	0.13–290.8	3(20%)	3(20%)	(Zhao et al. 2011; Hao et al. 2012; Liu et al. 2012a; Wang 2016)
*Folium isatidis*	2	1(50%)	1.2	0	0	2	1(50%)	1.2			(Hao et al. 2012)
*Mangnolia officinalis*	4	1(25%)	17.14	1(25%)	1(25%)	3	1(33%)	1.52			(Li and Zhuang 2000; Tan et al. 2012)
*Semen juglandis*	20	1(5%)	0.58	0	0						(Guo et al. 2012; Li et al. 2014)
*Rhizoma bletillae*	11	1(9%)	8	1(9%)	1(9%)	1	1(100%)	8	1(100%)		(Guo et al. 2012; Xie 2016)
*Colla corii asini*	10	2(20%)	1.12–2.85	1(10%)	0	10	2(20%)	2.04–2.85			(Li et al. 2017b)
*Radix Puerariae*	40	5(13%)	0.751–9.9	3(8%)	1(3%)	40	5(13%)	0.751–13.5	3(8%)	2(5%)	(Han et al. 2012; Wang et al. 2014; Chien et al. 2018)
*Fritillaria*	29	2(7%)	1.6–10.06	1(3%)	1(3%)	29	2(7%)	2.5–10.06	1(3%)	1(3%)	(Han et al. 2012; Wang et al. 2013; Chien et al. 2018)
*Radix Notoginseng*	41	9(22%)	2.64–273.93	9(22%)	6(15%)	41	9(22%)	2.64–267.95	6(15%)	4(10%)	(Hao et al. 2012; Chen et al. 2015; Ying et al. 2018)
*Hibiscus sabdariffa*	28	1(4%)	3.11	1(4%)	0	28	1(4%)	3.11	0	0	(Liu et al. 2018)

**Figure 2.**
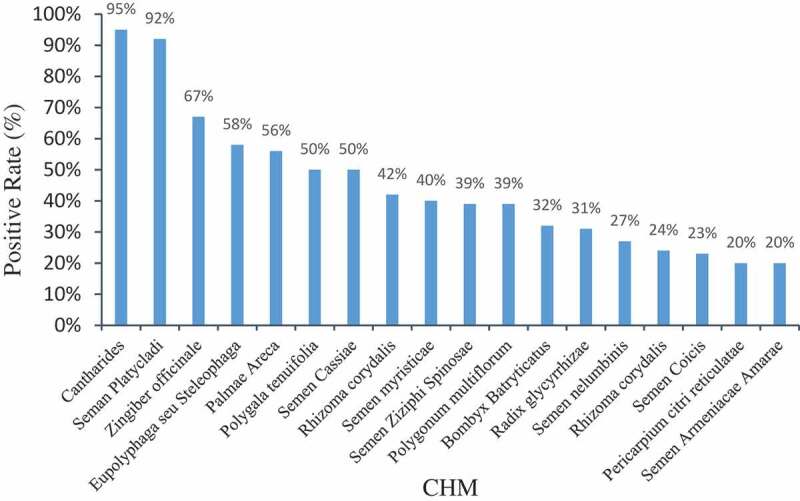
CHM with high AFs positive rate.

CHMs can be simultaneously contaminated by AFB_1_ and other AFs such as AFB_2_, AFG_1_, AFG_2,_ and AFM_1_. By analysing 2734 batches of CHM samples, the simultaneous occurrence of multiple AFs (AFB_1_ + AFB_2_ + AFG_1_ + AFG_2_ + AFM_1_) was detected to be 30% (Figure 2). Of the positive samples, there were 378 batches that exceeded the limit set by the EU and China (10 μg∙kg^−1^). In the case of botanicals, the incidence of AFB_1_ is higher than that of other AFs. However, AFG_2_ is the most prevalent AF contaminant found in certain herbal materials such as *Codonopsis Pilosula*, with contamination level as high as 471 μg∙kg^−1^ (Tan et al. 2012).

It is worth noting that among the 36 batches of animal material *Eupolyphagaseu Steleophaga* tested, 17 of 36 (47%) and 21 of 36 (58%) samples were found to be contaminated with AFB_1_ or multiple AFs, respectively. The occurrence rate of various AFs in different types of animal materials was not uniform. For example, AFG_1_ was the most commonly detected AF in the *Eupolyphagaseu Steleophaga* samples, with both a high occurrence rate and contamination level (Yang et al. 2011c; Liu et al. 2017; Sun et al. 2017; Luo et al. 2018), while in *Cantharides*, AFB_1_ is the most prevalent AF contaminant found (Sun and Liu 2016).

**Figure 3.**
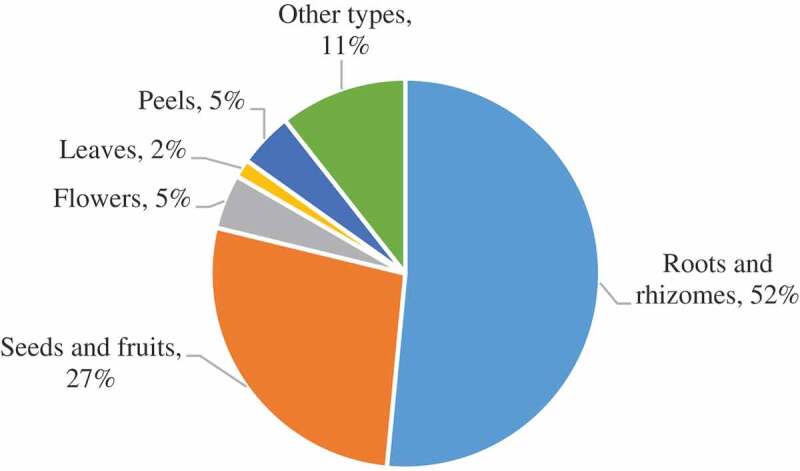
Detection of AFs in 66 CHM with different medicinal parts.

In addition, by analysing 66 types of CHMs, we found that the sample types most susceptible to AF contamination belong to different medicinal parts, including roots, rhizomes, fruits, and seeds (Figure 3). In addition, contamination of flower medicinal materials such as *Lilium brownii* (Zheng et al. 2014c) and *Lonicera japonica* (Cai et al. 2010) by AFB_1_ was detected at levels of 1.0 μg∙kg^−1^ and 50 μg∙kg^−1^, respectively. *Lonicera japonica* was easily contaminated by AFG_2_, and the contamination rate was 66.67%, with a highest detected contamination level of 203 μg∙kg^−1^ (Tan et al. 2012).

## 3. Ochratoxin

Ochratoxin is a type of mycotoxin mainly produced by *Aspergillus ochraceus, P. verrucosum,* and *A. carbonarius*. Ochratoxin A, ochratoxin B, ochratoxin C, and ochratoxin D are the main varieties of ochratoxins (Li and Ji 2003). Among the families of ochratoxin that have been discovered, ochratoxin A (OTA) is considered to be second after AFs in terms of prevalence and potential health hazards. OTA is carcinogenic, teratogenic, neurotoxic, and exposure can also result in hepatotoxicity and nephrotoxicity. Therefore, OTA is a mycotoxin and is classified as a Class IIB carcinogen by the International Agency for Research on Cancer (IARC) (Shu et al. 2008).

### 3.1. Regulatory guidelines for OTA levels

In the case of medicinal plants, the EU has official regulations on the level of OTA allowed in nutmeg, ginger, turmeric, black and white pepper, and liquorice root and its extract, with the legal limit varying from 15 μg∙kg^−1^ to 80 μg∙kg^−1^ (European Union 2006). In Vietnam, the limit for OTA levels ranges from 20 μg∙kg^−1^ to 80 μg∙kg^−1^ (United States Department of Agriculture Foreign Agricultural Service 2013) (Table 3).

**Table 3.** Limit of OTA for medicinal plants in Standards and Regulations.

Standards and regulations	Product (Group)	OTA (µg∙kg^−1^)	Reference
Vietnam	Nutmeg	30	(United States Department of Agriculture Foreign Agricultural Service 2013)
	Ginger and turmeric	20	
	Black and white pepper	80	
	Liquorice root used for herbal tea		
	Liquorice extract for beverage or to mix		
EU	Nutmeg	15	(European Union 2006)
	Ginger		
	Turmeric		
	White and black pepper		
	Liquorice root, ingredient for herbal infusion	20	
	Liquorice extract, for use in food in particular beverages and confectionary	80	

### 3.2. Detection methods for OTA

As described in previous studies, methods for detection of OTA include TLC, HPLC, ELISA, and GICA. Currently, HPLC-based methods are most commonly used for detection of OTA, with HPLC-MS/MS utilised often (Chen et al. 2011; Kuang and Qiu 2012b). In 2010, Yang et al. (2010) established the first HPLC-FLD method for detecting and quantifying OTA contamination levels in CHMs in China. Since then, HPLC-FLD has been routinely used to determine OTA levels in herbal medicines (Kuang et al. 2012a; Li 2015b). In 2010, Wu et al. also developed an HPLC with an ELSD (HPLC-ELSD) method for detection and quantification of OTA in CHMs (Wu et al. 2011a). The level of OTA present in 30 herbal medicines was determined via IAC sample purification, with a limit of detection (LOD) of 0.5 ng·g^−1^ and a recovery of 89.8%~94.6%.

Ultra-high performance liquid chromatography (UPLC) has also been successfully applied for analysing OTA levels in CHM (Cao et al. 2013; Yang et al. 2014b). A UPLC-based approach is more suitable for high-throughput detection of complex trace mixtures, since UPLC has the advantages of high sensitivity, high resolution, and a relatively short separation period (Zhang et al. 2018c).

Biological methods have been utilised as approaches for detection and quantification of OTA in CHMs. In 2015, Wang established a colloidal gold immunochromatographic method for rapid detection of OTA, and this approach is sensitive as low as 5 ng·mL^−1^ (Wang 2015). Zhou et al. developed an aptamer-based lateral flow strip relying on a competitive format that allows for rapid detection of OTA in *Astragalus membranaceus* (Zhou et al. 2016). After optimising some parameters, the aptamer-based assay demonstrated a visual LOD of 1 ng·mL^−1^. In the same year, Xiao et al. (2016) developed a rapid method for the detection of OTA in malt samples that is based on the indirect competition principle and flow microsphere technology.

### 3.3. OTA contaminants in CHMs

A total of 303 batches of Chinese herbal medicines (including 15 types of CHMs) were contaminated with OTA, with a per cent positive rate of 26% and a contamination range of 0.010–158.7 μg∙kg^−1^. Nineteen per cent of samples exceeded the EU set limit for OTA among the positive samples (Table 4). In the case of one type of CHM, OTA occurred in 4 out of 5 batches of *Glycyrrhiza uralensis* samples (Yang et al. 2010), and the highest contamination value was 84.4 μg∙kg^−1^.

**Table 4.** Detection of OTA in CHM.

	OTA				
CHM	Total samples	Positive samples No (%)	Range (µg∙kg^−1^)	>EU Legal limit No (%)	Reference
*Glycyrrhiza uralensis*	48	21(44%)	0.010–94.7	5(10%)	(Yang et al. 2010; Wei et al. 2011; Wang et al. 2013; Wei et al. 2013)
*Semen Armeniacae Amarum*	10	1(10%)	0.7	0	(Zheng et al. 2014c)
*Semen Pruni Persicae*	10	1(10%)	34.9	1(10%)	(Zheng et al. 2014a)
*Semen Plantaginis*	10	3(30%)	0.5–38.4	2(20%)	(Zheng et al. 2014c)
*Fructus Hordei Germinatus*	32	4(13%)	1.14–10.7	0	(Liu et al. 2013; Zheng et al. 2014c; Wang 2016)
*Fructus oryzae germinatus*	9	2(22%)	1.7–7.9	0	(Zheng et al. 2014c)
*Radix Ginseng*	10	10(100%)	0.04–5.86	0	(Bao et al. 2008)
*Zingiber Officinale Roscoe*	30	23(77%)	0.02–20.66	3(10%)	(Bao et al. 2008; Cao 2013)
*Astragalus Membranaceus*	3	3(100%)	87.7–158.7	3(100%)	(Yang et al. 2010)
*Massa Medicata Fermentata*	2	1(50%)	2.4	0	(Yang et al. 2010)
*Radix Notoginseng*	33	1(3%)	1.7	0	(Yang et al. 2010; Chen et al. 2015)
*Gossypium hirsutum* Linn.	1	1(100%)	27.1	1(100%)	(Yang et al. 2010)
*Alpinia oxyphylla*	44	1(2%)	6.59	0	(Zhao et al. 2017)
*Polygonum Multiflorum*	41	6(15%)	0.66–3.35	0	(Li et al. 2016)
*Radix Paeoniae* alba	20	1(5%)	0.53	0	(Xing et al. 2016)

**Figure 4.**
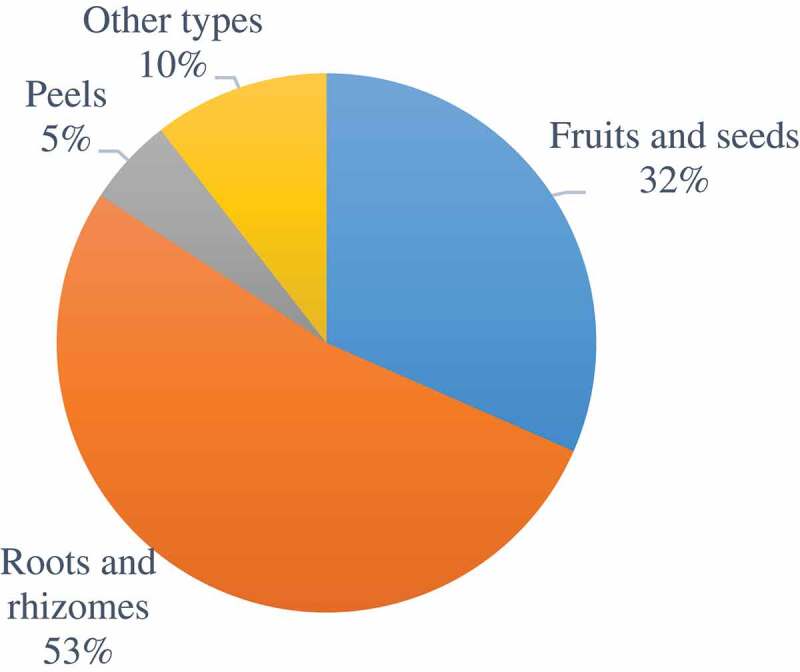
Detection of OTA in 19 CHM with different medicinal parts.

Roots, rhizomes, seeds, and the fruit of medicinal materials were susceptible to ochratoxin contamination, not unlike AF contamination in CHMs (Figure 4). The flower-based medicinal materials such as *Lilium brownie* (Zheng et al. 2014c) and *Urena lobate* (Yang et al. 2010), were found to be contaminated with OTA, and the contamination levels detected were 2.2 μg∙kg^−1^ and 1.7 μg∙kg^−1^, respectively.

## 4. Zearalenone

Zearalenone (ZEN) is an oestrogen-like mycotoxin produced mainly by *Fusarium graminearum* and *F. oxysporum*. Studies have shown that ZEN is a reproductive toxin and that exposure to ZEN has teratogenic effects. At concentrations of 1 nmol∙L^−1^–10 nmol∙L^−1^, ZEN can stimulate the transcription of oestrogen receptors and affect cell division and growth (Deng and Yuan 2007). ZEN is also able to cause DNA damage, inhibit protein and DNA synthesis, and interfere with the cell cycle to block DNA replication and inhibit cell proliferation; high doses of ZEN can induce damage to the immune system as well (Jiang et al. 2011).

### 4.1. Detection methods of ZEN

There are relatively few studies concerning the detection of ZEN in CHMs. At present, HPLC/MS/MS method is often used to analyse ZEN levels in herbal medicines (Tan et al. 2012; Zheng et al. 2014a).

Zhang et al. (2012) detected and quantified ZEN in 107 CHM samples using an HPLC with DAD (HPLC-DAD) method. Compared with an HPLC-FLD-based approach, this method has decreased sensitivity but can provide the chromatogram of ZEN, and also obtain the spectrogram of ZEN in positive samples. HPLC-FLD cannot obtain the spectrogram of a positive sample, which gives HPLC-DAD the advantage of increased ability to avoid detection of false positives. Wu et al. (2011b) proposed an HPLC-ELSD method, which could provide a convenient and reliable alternative to commonly used HPLC-FLD methods for the rapid determination of ZEN, as it uses a relatively simple, safe, fast, and cost-effective means for sample purification.

### 4.2. ZEN contaminants in CHMs

A study of the prevalence of ZEN contamination in 105 different CHMs revealed that 41 of them were contaminated by ZEN (per cent positive rate was 39%), and level of ZEN contamination ranged from 0.2 to 931.07 μg∙kg^−1^ (Table 5). Some reports have shown that seed fruits such as *S. Coicis* (Yang et al. 2011c; Kong et al. 2013) and *S. Persicae* (Han 2011) are easily contaminated by ZEN; in the case of *S. Coicis*, the ZEN-positive detection rate is as high as 83%. ZEN has been detected as a contaminant in roots, rhizomes, leaves, and in the case of one study, in the cortex of the herb *Juniperus procumbens* (Han et al. 2012), at a level of 2.3 μg∙kg^−1^. A very high level of ZEN was reportedly detected in *Cistanche tubulosa*, but due to the small number of samples, this finding bears additional exploration (Yang et al. 2011c).

**Table 5.** Detection of ZEN in CHM.

CHM	ZEN			Reference
	Total samples	Positive samples No (%)	Range (µg∙kg^−1^)	
*Semen Coicis*	18	15(83%)	23.3–931.07	(Yang et al. 2011c; Kong et al. 2013)
*Alpinia oxyphylla*	44	2(5%)	9.03–16.03	(Zhao et al. 2017)
*Radix Paeoniae* alba	27	13(48%)	0.7643–4.81	(Qin 2011; Xing et al. 2016)
*Folium Isatidis*	5	2(40%)	4.9958–20.1198	(Qin 2011)
*Rhizoma Corydalis*	1	1(100%)	1.4	(Yang et al. 2011c)
*Massa Medicata Fermentata*	1	1(100%)	0.2	(Yang et al. 2011c)
*Cistanche tubulosa*	1	1(100%)	271	(Yang et al. 2011c)
*Semen Pruni Persicae*	2	2(100%)	1.7–4.4	(Han 2011)
*Semen Armeniacae Amarum*	1	1(100%)	2.9	(Han 2011)
*Polygonum Multiflorum*	2	1(50%)	1.1	(Han 2011)
*Radix Scutellariae*	2	1(50%)	2.1	(Han 2011)
*Lygodium japonicum*	1	1(100%)	10.3	(Han 2011)

## 5. Other mycotoxins

Although AFs and OTA are the most commonly reported mycotoxin contaminants, occurrence of other mycotoxins such as fumonisins, trichothecenes, citrinin, and patulin has also been described in CHMs.

Fumonisin is a type of secondary metabolite produced by *F. oxysporum* and includes the A, B, C, P and FB_1_ derivatives. In 1993, it was classified as a Class B carcinogen by the International Agency for Research on Cancer. Fumonisin mainly damages the heart, liver, lungs, kidneys and other organs of animals, and exposure to fumonisin can result in porcine pulmonary oedema, liver damage, cardiovascular disease, and equine leukoencephalomalacia. In addition, exposure to fumonisin may cause human oesophageal cancer and neural tube defects (Yang et al. 2014a).

Trichothecenes is a class of secondary metabolites produced by different Fusarium species, such as *F. graminearum* and *F. serrata*; compounds in this group include T-2 toxin, deoxynivalenol (DON), nivalenol (NIV), diacetoxyscirpenol (DAS), and its derivatives (Yue 2009). Studies have shown that T-2 toxin is one of the most toxic mycotoxins among the type-A trichothecene mycotoxins. T-2 toxin can inhibit the synthesis of cellular proteins, DNA and RNA, trigger DNA damage via oxidative stress, induce apoptosis, alter gene expression patterns, and damage the cell membrane. T-2 toxin can also cause pathological changes in liver tissue and damage to the immune system (Zhou et al. 2011). DON, also called vomiting toxin, is highly cytotoxic, induces apoptosis, inhibits proliferation of immune cells, induces cytokine production from helper T-cells, and activates macrophages and T-cells, resulting in additional cytokine production (Huo et al. 2008).

Citrinin is a mycotoxin produced by filamentous fungi including *Penicillium, Aspergillus,* and *Monascus*. As a nephrotoxin, citrinin exposure can cause kidney disease in a variety of animals such as dogs, pigs, rats, chickens, and birds. Citrinin exposure can also induce mutations and result in deformities and tumours (Li et al. 2011b). Furthermore, the effects of citrinin can synergise with other mycotoxins (such as ochratoxin and patulin) to inflict more deleterious effects to tissues and organs (Liu and Xu 2004).

Patulin is a genotoxic compound that has been found to have broad toxicity and exposure to patulin can cause a variety of symptoms in humans and animals, including nausea, vomiting, blood in the stool, convulsions, and coma (Zhou et al. 2010). In addition, patulin exposure can result in acute and subacute poisoning. Furthermore, exposure to patulin has been reported to have cytotoxic, teratogenic, carcinogenic, and immunotoxic effects.

### 5.1. Detection methods for other mycotoxins

Fumonisin is currently detected using HPLC-MS/MS. In 2011, the method for simultaneously detecting fumonisin B_1_ (FB_1_) and fumonisin B_2_ (FB_2_) in 34 types of CHMs was developed by Xie et al. (2011). An immunoaffinity column was used to purify samples and the detection limit for FB_1_ and FB_2_ with this approach was 2 μg∙kg^−1^.

A method to detect T-2 toxin contamination in CHMs using GC with ECD (GC-ECD) was first proposed by Yue et al. (2009). In order to improve the selectivity and sensitivity of the method, sample clean-up was performed using an immunoaffinity column, and N-(heptafluoro-n-butyl) imidazole (HFBI) was then used for pre-column derivatisation. The per cent recovery of starting material ranged from 82.2% to 98.6%, and the LOD was 2.5 μg∙kg^−1^. Subsequently, the same authors established a method to detect DON in CHMs and related products using GC-ECD. Application of this approach showed that the per cent recovery of various CHM starting material ranged from 85.5% to 97.2%, the detection limit for DON with the method was 2.0 μg∙kg^−1^. This is the first report on the detection of DON contamination in CHMs and related products (Yue et al. 2010a).

In 2011, Wang et al. (2011) detected patulin in *Fructus Aurantii* by HPLC-MS/MS. A few years later, Zhou et al. (2015) established HPLC-DAD method for the analysis of patulin in *F. Crataegi*. In this study, a home-made solid-phase extraction (SPE) column was prepared using self-made poly-vinylpyrrolidone-Flory silica (PVPP-F) as sorbent for sample pre-treatment, and the detection limit of the method was 3.56–3.99 μg∙kg^−1^.

### 5.2. Other mycotoxin contaminants in CHMs

Mycotoxins such as FB, T-2 toxin, and DON have been successfully detected in CHMs (Table 6). For example, the fruit and seeds of CHMs such as *S. Sterculiae Lychnophorae* and *S. Coicis* are susceptible to fumonisin B contamination.

**Table 6.** Detection of other mycotoxins.

CHM	Detection Methods	Maximum Contamination Values of Other Toxins (μg∙kg^−1^)				References
		FB_1_	FB_2_	T-2	DON	
*Semen Armeniacae Amarum*	UPLC-MS/MS	0.89	1.65			(Han 2011)
*Radix Paeoniae* alba	UPLC-MS/MS			0.69		(Han 2011)
*Mangnolia Officinalis*	HPLC-MS/MS	397	793			(Xie et al. 2011)
*Astragalus Membranaceus*	HPLC-MS/MS		158			(Xie et al. 2011)
*Radix Puerariae*	HPLC-MS/MS			1.095		(Chen 2012)
*Polygonum Multiflorum*	UPLC-MS/MS	2.57	1643.2	1.93		(Li et al. 2016)
*Semen Pruni Persicae*	UPLC-MS/MS HPLC-MS/MS	82.3	18.9		803.4	(Han 2011; Zheng et al. 2014a)
*Fructus Forsythiae*	HPLC-MS/MS	29.4	7.8			(Ge et al. 2011)
*Scutellaria Baicalensis*	HPLC-MS/MS	6.7	208			(Xie et al. 2011)
*Panax Notoginseng*	UPLC-MS/MS			0.258		(Chen et al. 2015)
*Semen Sterculiae Lychnophorae*	HPLC-MS/MS	125	2240			(Xie et al. 2011)
*Semen Coicis*	HPLC-MS/MS		562	167		(Xie et al. 2011)
*Lysimachia nummularia*	UPLC-MS/MS	2.50	1.25			(Han 2011)
*Radix Asparagi Cochinchinensis*	HPLC-MS/MS	79.4	173			(Xie et al. 2011)
*Radix Isatidis*	HPLC-MS/MS	23.8	126			(Xie et al. 2011)
*Medicinal Fermented Mass*	HPLC-MS/MS	113	90			(Xie et al. 2011)
*Rhizoma Dioscoreae*	UFLC-MS/MS	3.727				(Li 2016)
*Folium Isatidis*	UPLC-MS/MS	3.80	0.78			(Han 2011)
*Radix Salviae Miltiorrhizae*	UPLC-MS/MS			0.3		(Han 2011)
*Lonicera Japonica*	UPLC-MS/MS			0.2		(Han 2011)
*Radix Paeoniae Rubra*	UPLC-MS/MS			0.4		(Han 2011)

Xie et al. analysed 34 types of CHM samples and found 11 fumonisin-positive samples, with fumonisin concentrations ranging from 82.4 to 2349 μg∙kg^−1^ (Xie et al. 2011). In the same year, the contamination level of FB_1_ and FB_2_ in some CHMs was determined by Han (2011). The analysis revealed that the range of FB_2_ in *S. Sterculiae Lychnophorae* was 928–2240 μg∙kg^−1^, and the highest detected levels of FB_1_ and FB_2_ in *S. Coicis* were 562 μg∙kg^−1^ and 167 μg∙kg^−1^, respectively. Notably, it was found that FB_1_ and FB_2_ were usually detected in samples together, although the contamination levels for the two mycotoxins were rarely similar. For example, the incidence of FB_2_ contamination in the roots and rhizomes of *Polygonum multiflorum* (Li et al. 2016) was as high as 1643.2 μg∙kg^−1^, while FB_1_ was only detected at a level of 2.57 μg∙kg^−1^. Occurrence of T-2 and DON contaminations has been reported in several CHMs to date. Zheng et al. analysed mycotoxin content in the fruit and seeds of the CHMs *S. PruniPersicae* and *S. Coicis*. The results showed that the highest level of DON detected in *S. PruniPersicae* was 803.4 μg∙kg^−1^, but DON was not detected in the *S. Coicis*; T-2 was not detected in either case (Zheng et al. 2014c). The highest level of T-2 detected in the rhizomes of CHMs such as *R. Paeoniae* alba (Han 2011), *R. Salviaemiltiorrhizae,* and *R. Notoginseng* (Chen et al. 2015) was less than 0.7 μg∙kg^−1^, indicating that at least some CHMs are not easily contaminated by T-2.

## 6. Detection of multiple mycotoxins

There are often more than one type of mycotoxin contaminants present in CHMs. Thus, it is important to consider the possibility of and test samples for multi-mycotoxin contaminants. For example, fruit and seeds from CHMs such as *S. Armeniacae Amarum* (Cai et al. 2010; Han 2011; Han et al. 2011; Zheng et al. 2013; Zheng et al. 2014c; Zhao et al. 2016) *S. Coicis* (Cai et al. 2010; Zheng et al. 2010b; Xie et al. 2011; Kong et al. 2013; Liu et al. 2013), *S. Persicae* (Cai et al. 2010; Han et al. 2010; Zheng et al. 2010b; Han 2011; Han et al. 2011; Li et al. 2011a; Zheng et al. 2014a) and *S. Sterculiae Lychnophorae* (Cai et al. 2010; Xie et al. 2011; Su et al. 2014) are not only susceptible to AF contaminants but are also often co-contaminated with other mycotoxins such as OTA, ZEN, and FB.

Along with a variety of methods for detecting different mycotoxins, methods for simultaneous determination for various mycotoxins have been gradually developed. AFB_1_ and OTA contaminants in CHMs can be detected together by HPLC-FLD, with use of a composite immunoaffinity column for sample cleanup (Wei et al. 2011; Cao et al. 2013). Furthermore, simultaneous detection of DON and NIV in CHMs by HPLC-UV was first proposed by Yue et al. (2010b). The sample pre-treatment procedure used in this work abolished the derivatisation step used in the conventional approach to yield improved results. Several years later, Kong et al. (2012) developed a method for simultaneously measuring T-2 and HT-2 toxins in CHMs.

With the spread of modern MS technology, new methods for the combined detection of mycotoxins with large chemical diversity continue to be developed and applied. At present, it has been demonstrated that up to 35 different toxins can be detected from an herbal matrix in a single HPLC-MS/MS run (Han et al. 2012).

## 7. Conclusion

At present, mycotoxin contaminants have become some of the most prevalent hazardous substances in CHMs and a major public safety concern regarding their sale and use. In this review, we summarised some common mycotoxin contaminants found in medicinal materials and discussed methods for their detection. Mycotoxin contamination is usually heterogeneous, so screening methods for detecting these contaminants in medicinal products need to have broad coverage across, and samples should be processed carefully (Zhang et al. 2018a; Tittlemier et al. 2019). However, many existing studies of mycotoxin contamination lack detailed descriptions of how samples are selected and processed. To guarantee an accurate snapshot of any existing mycotoxin contaminants, careful considerations need to be taken with regard to sample acquisition and processing. Another complication in collecting accurate data lies in that the rapid detection methods such as ELISA and GICA are more prone to false negatives or false positives than conventional detection methods. Therefore, further validation should be performed on any significant findings that rely on rapid detection methods. In addition, the mycotoxin contamination is a known problem in Chinese herbal medicines. For example, *S. Platycladi* and *S. Ziziphi Spinosae* are prone to aflatoxin contamination, *Glycyrrhiza uralensis* and *Zingiber Officinale Roscoe* are prone to OTA contamination, and ZEN contamination is prevalent in *S. Coicis* and *R. Paeoniae alba*. In some medicines, s*uch as S. Persicae a*nd *Polygonum multiflorum*, co-occurrence of multiple mycotoxin contaminations had been detected.

Although some producers of CHMs currently have some standards in place for monitoring the levels of mycotoxins such as AFs and OTA, standardised guidelines regarding monitoring for other mycotoxins and their levels have not been established for CHMs. Therefore, it is necessary to conduct additional research to better understand which CHMs are easily contaminated by which mycotoxins. This information can then be used to establish guidelines for screening for mycotoxin contaminants and limitations on acceptable levels in CHMs.

Rapid analytical methods for mycotoxin detection are currently under development and are increasingly utilised by CHM producers. In recent years, standard biological analysis methods have been utilised for detection of mycotoxins in CHMs; such methods include the GCIA and ELISA approaches. Novel technologies such as ultrasensitive mycotoxin biosensors have been developed and utilised for mycotoxin screening in food and serum. For example, Taghdisi et al. (Taghdisi et al. 2016) developed a fluorescent aptamer sensor (aptasensor) that allowed for selective and sensitive detection of OTA in grape juice and serum. Since then, this group has proposed another accurate fluorescent sensing method for the determination of AFB_1_ in grape juice and human serum samples based on a hairpin structure of a G-quadruplex oligonucleotide-aptamer chimera (Taghdisi et al. 2018). A highly sensitive aptasensor utilising the fluorescence resonance energy transfer for AFM_1_ detection in milk samples was recently developed (Li et al. 2017a). Another group established a surface plasmon resonance (SPR) method using an SPR sensor chip for simultaneous detection of AFB_1_, OTA, ZEN, and DON in corn (Wei et al. 2019). However, broad application of these recently developed mycotoxin detection methods in CHMs requires further validation.

In recent years, studies on the presence of mycotoxin contaminants in CHMs have mainly focused on identifying the varieties of mycotoxins present, determining the contamination level, and refining mycotoxin detection methods. In contrast, relatively few studies have examined these mycotoxin contaminations in the context of toxigenic mechanisms, detoxification techniques, and prevention and control measures. The presence of specific mycotoxin contaminants and their relative abundance in medicinal materials is intimately related to the place of origin, processing methods, and storage conditions. Therefore, future studies should focus on investigating the occurrence of mycotoxin contamination in CHMs in various storage conditions, and findings from these studies can be used to help establish an efficient prevention strategy to minimise the presence of fungi and mycotoxin contaminants in CHMs.
